# A Critical Role for Network Structure in Seizure Onset: A Computational Modeling Approach

**DOI:** 10.3389/fneur.2014.00261

**Published:** 2014-12-08

**Authors:** George Petkov, Marc Goodfellow, Mark P. Richardson, John R. Terry

**Affiliations:** ^1^College of Engineering, Mathematics and Physical Sciences, University of Exeter, Exeter, UK; ^2^Institute of Psychiatry, King’s College London, London, UK

**Keywords:** network dynamics, epilepsy, dynamical systems, graph theory, EEG

## Abstract

Recent clinical work has implicated network structure as critically important in the initiation of seizures in people with idiopathic generalized epilepsies. In line with this idea, functional networks derived from the electroencephalogram (EEG) at rest have been shown to be significantly different in people with generalized epilepsy compared to controls. In particular, the mean node degree of networks from the epilepsy cohort was found to be statistically significantly higher than those of controls. However, the mechanisms by which these network differences can support recurrent transitions into seizures remain unclear. In this study, we use a computational model of the transition into seizure dynamics to explore the dynamic consequences of these differences in functional networks. We demonstrate that networks with higher mean node degree are more prone to generating seizure dynamics in the model and therefore suggest a mechanism by which increased mean node degree of brain networks can cause heightened ictogenicity.

## Introduction

Epilepsy is a serious neurological disorder characterized by the propensity of the brain to generate spontaneous and recurrent seizures. Traditionally, seizures have been defined as “a transient occurrence of signs and/or symptoms due to abnormal, excessive, or synchronous neural activity in the brain” (1). Very recently, the international league against epilepsy (ILAE) has further refined the definition of epilepsy (2) whereby an individual is now proposed to have epilepsy if one of the following conditions is met:

1. Experiencing two unprovoked seizures more than 24 h apart.
2. Experiencing a single unprovoked (or reflex) seizure with a probability of further seizures similar to the general risk of recurrence (~60%) if two unprovoked seizures had occurred.
3. An epilepsy syndrome is diagnosed.

It is important to note that epilepsy is a general term to capture over forty, often diverse, syndromes. However, in each case, the generation of clinical signs and symptoms are presumed to require large regions of the brain to be subject to abnormal dynamics and the initiation, recruitment, and spreading of such dynamics is facilitated by the network of synaptic connections between neurons and between regions of the brain. This is reflected in the recognition of the ILAE that many epilepsy syndromes are associated with disruptions to either global or local brain networks (3).

However, a precise definition of global and local brain networks is surprisingly non-trivial. In the global case, one can consider large-scale structural networks as defined by white matter tracts of axons that connect distal brain regions. These networks can be estimated non-invasively using diffusion imaging. An alternative is to examine the statistical inter-relationship between time series recorded at different locations in the brain, thus, defining a “functional” rather than a structural network. While to some extent, functional networks are constrained by the structural architecture of the brain, they also carry contributions from the dynamics of brain activity (4). We recently studied functional networks derived from scalp electroencephalogram (EEG) at rest and demonstrated significant differences between functional networks of people with idiopathic generalized epilepsy (IGE), their first-degree relatives, and healthy controls (5). Significant differences across a number of graph theory measures highlighted abnormalities in both the epilepsy cohort and their first-degree relatives. The most significant of these was that the mean node degree of networks inferred from both people with IGE and their relatives was much greater than that of controls, but that no differences were found between patients and their relatives. This observation suggests that differences between patients and controls cannot be attributed to medication, and thus, altered functional networks are associated with a propensity to generate recurrent seizures (i.e., epilepsy). However, abnormalities in these networks alone are not sufficient to generate seizures (since they are present in the relatives of people with IGE, whom themselves are seizures free) suggesting that the interplay between functional network structure and the dynamics supported by them must play an important role in seizure generating capability (ictogenicity).

The use of mathematical modeling to attempt to address this and related questions has grown substantially in the past few years. Particularly at the macroscopic scale, where the average response of a mass of neurons is represented by systems of differential equations, several studies have derived insight into the potential dynamic mechanisms that enable seizures associated with spike-wave discharges to emerge spontaneously from background activity (6–10). Lopes da Silva et al. (11) proposed a scenario in which the spontaneous transitions between background activity and seizure states arise due to bistability, i.e., that the background state and seizure state “coexist” and random inputs can perturb the brain from one state to another. This can be interpreted in terms of either state being able to be reached without a change in underlying constants or slowly varying parameters of the system. This type of model was used to demonstrate that the emergence of either focal or generalized seizure like events could occur due to either specific network disruptions or to alterations in excitability within apparently normal network structures (12).

Motivated by a desire to understand the fundamental mechanisms of seizure transitions more clearly, the concept of bistability has formed the basis of more abstract models of the brain, for example, the so-called Z^6^ model (13), which provides a phenomenological representation of the critical features of more realistic physiological models. These abstract models, which we might consider to represent a normal form of the more detailed physiological representations, have recently been extended to study the role that explicit network structures have in facilitating transitions into seizure activity (14, 15).

Here, we build on this previous modeling work to further understand the role of network topology in the generation of transitions into seizure dynamics. In order to understand the potential consequence on ictogenecity of the differences in network structure highlighted by Chowdhury et al. (5), we artificially construct networks that preserve the values of mean node degree for each subject. When these networks are used as the connectivity structure for a bistable dynamic network model, we observe that networks with higher mean node degree transition more readily to a seizure state. We therefore suggest a mechanism by which increased mean node degree of brain networks can cause increased ictogenicity.

## Materials and Methods

### Mathematical model

Since we focus on the role that network structure plays in transitions between background and seizure states, we do not consider a detailed model of each node in a network. Instead, the foundation of our present work is a network of abstract models that are designed to capture a bistable transition between a “background” state and a high-amplitude “seizure” state [see, e.g., Kalitzin et al. (16)]:

(1) $$\frac{d}{\mathrm{dt}}Z=\left(a|Z{|}^{4}+b|Z{|}^{2}+C\right)Z+\varepsilon \left(t\right),$$

where *Z* = *x* + *iy* is a complex variable (function of time); (*a*, *b*) are real constant coefficients, and *C* = *c* + *i*ω is a constant complex coefficient. The term ε(*t*) is the complex input to the system, which incorporates a white noise component to mimic the effects of exogenous fluctuations.

A network model, where each node has as its basis the system described in Eq. 1 is then constructed:

(2) $$\frac{d}{\mathrm{dt}}Z_{i}=\left(a|Z_{i}{|}^{4}+b|Z_{i}{|}^{2}+c+i\omega \right)Z_{i}+\sum_{j=1}^{N}G_{\mathrm{ij}}Z_{j}+\varepsilon_{i}\left(t\right)$$

Here, we consider the dynamics of *N* units, with linear interaction through an adjacency matrix *G*, where white noise is generated independently for each node within the network. In the current work, *G* is scaled by a factor of 0.1 to preserve transitions between states.

Model parameters are based upon our previous work (16) so that each node lies within the bistable regime. This allows transitions to occur between the steady state (SS), and limit cycle (LC) attractors, where the LC is considered to represent seizure dynamics.

### Clinical EEG recordings and construction of functional networks

The network measures that form the basis of this study were inferred from clinical EEG recordings as described in Ref. (5). In brief, these recordings consisted of 19 channel scalp EEG obtained using standard 10–20 placing with an average reference, and sampled at 256 Hz. The recordings were band-passed between 1 and 70 Hz, and notch-filtered between 48 and 52 Hz to exclude mains frequency interference. The subjects from whom the EEG recordings were taken are divided into two main groups: 35 people with heterogeneous IGE and 40 healthy controls. From each EEG recording, one artifact-free, eyes-closed, 20 s segment was extracted representing a “resting state” or “background” EEG activity. Chowdhury et al. (5) found significant differences between controls and patients in the 6 and 9 Hz “low alpha” frequency band, and we therefore focus on that band here. The Hilbert transform was applied to the band-pass filtered EEG to generate instantaneous phase and amplitude estimates. For each electrode pair, the phase-locking factor [PLF, also known as phase-locking value (17) or mean phase coherence (18)] was calculated as follows:

(3) $$C_{1}=c_{\mathrm{ij}}=\frac{1}{N_{s}}\left|\sum_{k=1}^{N_{s}}e^{i\Delta \phi_{\mathrm{ij}}\left(t_{k}\right)}\right|$$

where Δϕ*_ij_*(*t_k_*) is the instantaneous phase difference between signals *i* and *j* at the time point *t_k_*. The Δϕ*_ij_*(*t_k_*) were reconstructed from the original signals using the Hilbert transform.

This yields a value between 0 and 1 reflecting the strength of synchronous activity between each pair of signals. Functional networks were then constructed using electrode locations as nodes and PLF values as connectivity weights. Since the PLF measure is symmetrical, the resulting functional connectivity networks are undirected.

### Network measures

The derived functional networks were quantified using the following graph theory measures: mean degree (MD), degree variance (DV), and local clustering coefficient (CC). The degree of a node is defined as the sum of the weights of the edges incident to that particular node. The MD and DV are defined as the average and the variance, respectively, of degrees over all nodes in the network. The local CC of a node in a network measures how close its neighbors are to a complete network (graph).

### Generation of artificial networks

We note that the networks used for connectivity in the model in this study were not directly inferred from patient data, rather “surrogate” networks were prepared, which preserved properties of the networks studied in Chowdhury et al. (5). Each matrix was originally based on the functional connectivity matrix inferred from the aforementioned EEG data. An undirected binary network with the equivalent value of MD as the original matrix was constructed by applying a set of thresholds to the original, and choosing the threshold for which MD was preserved. Further a computational algorithm was applied (19) in order to randomize the matrix, preserving the degree vector and therefore the MD value. In brief, the algorithm randomly swaps nodes and recalculates the degree vector, checking for disparity. For each original matrix, we constructed 30 artificial random binary matrices with the same MD value as the original weighted connectivity matrix. We verified that the spectrum of the artificial patient and control derived networks was different, confirming a difference in topology of the artificial networks.

### Measure of brain network ictogenicity

We measured the “ictogenicity” of each network by performing simulations using the network as the connectivity matrix for the mathematical model. Since we calculated this measure of ictogenicity from model simulations, we could define an appropriate model state that captured transitions between the non-seizure and seizure dynamics of the model. In the current work, the model seizure state was defined as a solution with local maxima and minima having magnitude >0.5.

For each simulation, of the model Eq. 2, we calculated the time that each node spent in a *LC*, normalized to the simulation time. Averaging over all the nodes, we obtain the probability of any node to be in a *LC and we refer to this probability as the brain network ictogenicity* (BNI).

### Statistical analysis

For comparison of quantitative network measures between groups, we used a non-parametric Kruskal–Wallis one-way ANOVA test. Results are declared significant for *p* < 0.05. For *post hoc* pairwise comparisons between groups, a Bonferroni corrected multiple comparison test was performed with significance level of 0.05.

## Results

### Functional networks

**Figure 1:**
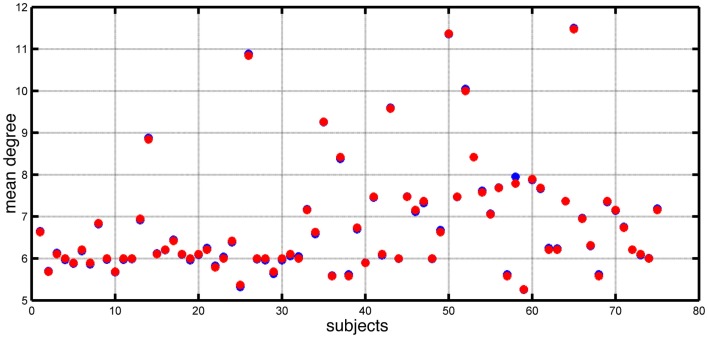
**Mean node degree values for each subject (blue dots) and each corresponding artificially constructed binary network (red dots)**.

Chowdhury et al. (5) reported that the MD of functional networks derived from people with epilepsy was higher than controls. In Figure 1, we show the distribution of MD for both epilepsy and control subjects included in that study. In this study, we focus on the dynamic consequences of changes in node degree independent of specific network topology and connectivity weights. We remove a layer of complexity from these networks by transforming them into binary (unweighted) networks, while preserving the MD of networks extracted from the EEG data. Figure 1 demonstrates the match in value of mean node degree between the original networks and the artificially derived alternatives (see Materials and Methods).

**Figure 2:**
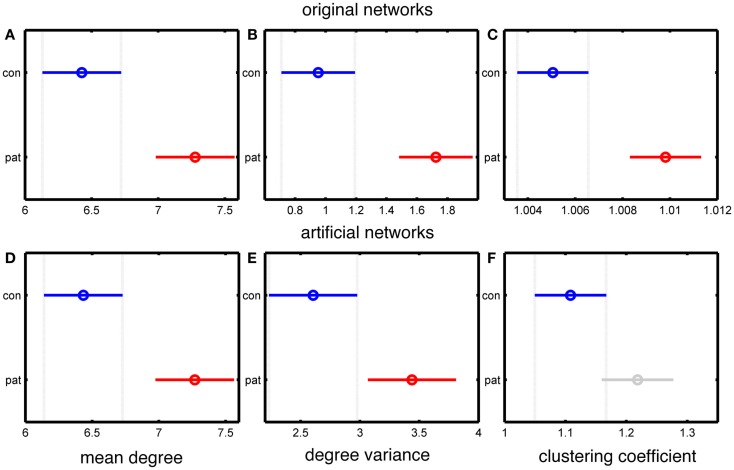
**Statistical analysis of the differences between the group mean values of people with epilepsy and healthy controls based on the MD, DV, and CC measures of the connectivity matrices**. **(A–C)** represent data from original networks, whereas **(D–F)** represent data for artificial networks. **(A,D)** show MD, **(B,E)** show DV, and **(C,F)** show CC. The *y*-axis of each panel separates the two groups (control and patients), and the *x*-axis represents the group values of the corresponding network measure. The results are color coded blue for the control group and red for the epilepsy group (except for panel **F**, in which the epilepsy group is colored grey to indicate a lack of statistically significant difference). The horizontal line and the circle show the variance and the mean value of the corresponding network measure. The mean values are considered as statistically significant different if there is no overlap between the lines within a panel.

Since the MD is accurately preserved in our artificial networks, the significant difference in MD between patients and controls is also maintained, as shown in Figure 2. The use of binary, rather than weighted networks leads our artificial networks to have higher DV than the original networks, as demonstrated in Figures 2B,E. A further reason for this difference is that the networks in Chowdhury et al. (5) were normalized to the DV value of 500 surrogate random networks, while in the present case of binary networks such normalization is not possible. However, Figures 2B,E show that a significant difference in DV between epilepsy and control subject derived networks is preserved.

Figures 2C,F demonstrate a lack of significant difference in CC between artificial “control” and “epilepsy” networks, in contrast to the EEG derived networks. This demonstrates that our artificially generated networks have removed some specific topological features of the original data, including those related to clustering.

### Modeling results

**Figure 3:**
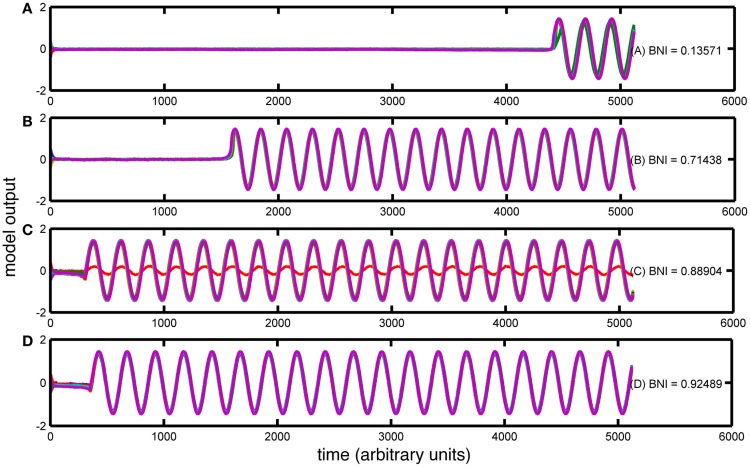
**Trajectories of four simulations with the Z^6^ model over different artificially created networks**. The figure contains **(A–D)** four simulations with different BNI values (as indicated). The *x*-axis of each panel represents the simulation time (arbitrary units) and the *y*-axis represents the amplitude of the simulated signal. In each case, all 19 channels are overlaid in different colors.

For each value of MD extracted from the epilepsy and control cohorts, 30 artificial networks were generated, preserving the MD. These networks were used as the connectivity scheme in the bistable model as described in Section “Materials and Methods.” For all simulations of our network model Eq. 2, we fixed model parameters corresponding to the bistable phase space of a single node {*a*, *b*, *c*, ω} = {−1, 2, −0.9, 1 + δω}, where δω is a random number distributed equally in the interval [−0.2, 0.2]. This choice is made to avoid artificial phase locking because of the equal phase velocities within our multi-unit configuration. For each network, 30 simulations were performed with random initial conditions. The resulting dynamics were quantified according to the BNI measure described in Section “Materials and Methods.” An example of the calculation of BNI and the effect of changes in node degree is given in Figure 3.

Four different kinds of dynamics can be seen in Figure 3. In Figure 3A, the model spends a large portion of the simulation time in the “background” attractor before transitioning to the seizure state. Thus, the BNI measurement is low. In contrast, the trajectory of the model in Figures 3B–D moves more quickly into the “seizure” attractor, and so BNI is higher. In addition, in Figure 3C, one of the nodes has not transitioned to the trajectory corresponding to the LC attractor in a single node. Rather, this node is being driven around the corresponding fixed point and therefore the BNI in this case is lower. It is clear that in this model, BNI provides a measure of how quickly the trajectory of the system performs an “escape” from the background to the seizure attractor.

**Figure 4:**
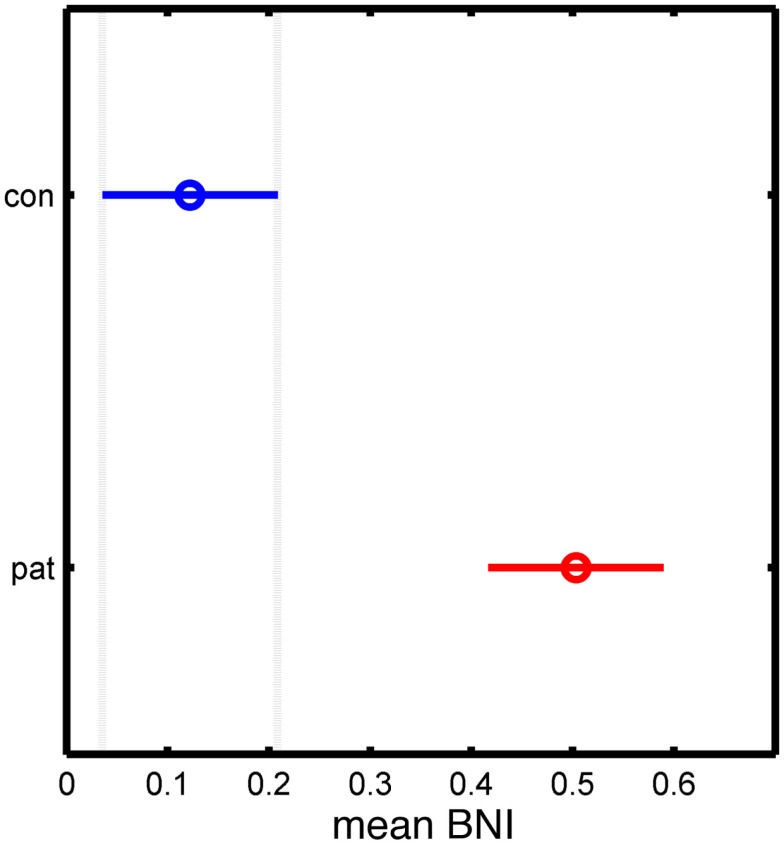
**Statistical analysis of the significant differences between control and patient groups based on the mean BNI measure**. The *y*-axis separates the two groups, while the *x*-axis represents the mean BNI value. The horizontal line and the circle represent the variance and the mean value of BNI, respectively.

Figure 4 shows BNI calculated from all simulations for artificial networks derived from the patient and control networks. It can be seen that BNI is significantly higher in the patient versus the control networks, and thus, networks with an increased node degree are shown to have a greater tendency toward seizure activity in this model.

## Discussion

In this study, we used mathematical modeling to investigate the link between the structure of brain networks and their propensity to generate seizure dynamics. Building upon previous studies, we used human EEG data to generate artificial networks preserving MD values, and thus, “isolating” this property for investigation. When networks with high MD were used as connectivity matrices in a model of seizure transitions, we observed significantly more time in the seizure state, as compared to networks with lower MD. We therefore provide evidence for a link between certain properties of network structure (here the MD) and the potential to generate seizure dynamics.

From the network perspective, MD and DV reflect how well connected the nodes within a graph are. Thus, networks with high MD and low DV would tend toward being fully connected, whereas networks with high MD and high DV will have an increased number of “hub-like” nodes. Our randomized networks in the patient group displayed higher MD and DV than controls and therefore fall predominantly into this latter category. This suggests that “hub-like” nodes can more easily drive the rest of the network into the seizure state if they themselves enter that state.

Previous modeling studies in the context of temporal lobe seizures and the hippocampus have suggested a role for hub-like connectivity in generating hyper-excitability (20). Such structures have also been shown to be critical for dementia (21), a condition with which epilepsy is comorbid (22), as well as other pathologies of the brain (23). In a related study, Clemens et al. (24) performed a resting EEG derived, functional connectivity network analysis of people with juvenile myoclonic epilepsy (JME) and control subjects. They found no statistically significant differences in measures of local and global efficiency of the derived networks, where “efficiency” relates to the length of the shortest paths between nodes. We should therefore aim to elucidate exactly which topological features of networks can contribute to the generation of seizure dynamics. In future work, we will explore in more detail the dynamic role of centrality, efficiency, and other features of network topology (25) on seizure generation in our model.

The model employed in this study provides an abstract representation of the epileptic brain. It preserves the potential for transitions between “background” and “seizure” dynamics as postulated in the bistable perspective of generalized seizures (11). This simplified approach allows one to focus upon the role that network structure plays in the propensity for dynamic transitions. Indeed, this approach has been used with success in terms of estimating transition frequencies (26), exploring the key dynamic components for intermittent transitions (15) and examining the role of specific connection topologies in small networks (14). An interesting extension to the current work would be to assess the interplay between intrinsic node dynamics and network structure. This could be achieved by using abstract models with richer bifurcation structures (15, 27), or by employing neural mass models of specific epileptiform dynamics (8, 10, 28, 29).

We built artificial networks preserving MD so as to focus on the implications of changes in this property, with respect to the process of transitions from SS to LC. Precise analysis of the model Eq. 2 leads to the conclusion that the behavior of the system in these terms may depend on several factors such as (a) noise level, (b) initial conditions, (c) connection strength, and (d) network topology. As the main goal was to examine the influence of network topology, we removed the influence of all other factors by setting appropriate noise levels, randomly sampling initial conditions, and using binary instead of weighted networks. In future work, we will consider the effects of adding larger variance noise into the model, in order to facilitate recurrent transitions. In addition, we can expand upon the approach by analyzing weighted networks. We envisage that the addition of these kinds of heterogeneities will lead to a richer repertoire of model dynamics, and therefore, might be useful in further stratifying the effect of network topology on dynamic transitions.

Benjamin et al. (14) examined escape times into seizure dynamics in a similar model applied to networks with a small number of nodes. In that case, it was possible to derive analytic expressions for escape times depending on the topology of networks. However, the complexity of this problem grows significantly as larger networks are considered. Here, in order to link directly with clinical data, we used a model with 19 nodes to represent EEG sensor space. Rather than focusing on explicit network structure, we were able to correlate changes in BNI with properties of the network, e.g., the MD. This provides an avenue to explore the seizure generating potential of more complex networks and could be extended in future work to include other graph theoretic measures, such as the CC, which has also been shown to vary significantly between people with IGE and controls (5, 27).

We used functional connectivity as the basis for the networks applied to our model, reflecting the nature of the available clinical data. This approach means that our model is not a direct representation of brain regions interacting over large scales via axonal connections, though such a model can be built in a patient specific way using diffusion data (29, 30). Rather, our model provides an abstract representation of the resting state of the brain, as projected onto the level of EEG. Networks derived from this projection are thought to be constrained by structural connectivity, though they are not a direct reflection of it (4). Functional networks by definition represent nodes that are evolving similarly, and therefore, capture a potentially important means by which information can be exchanged between brain regions (31). We should therefore consider that the “connections” of such networks can facilitate the emergence of pathological dynamics through synchronization, and we demonstrate here that this can lead to greater seizure generating potential in the epileptic brain.

On the other hand, functional networks can be viewed more simply as transformations of time series data recorded from subjects, i.e., as mappings from multivariate time series onto a static topological network that reflects a combination of structural and dynamic contributions for that instance of time. From this perspective, our modeling approach gives us a tool with which to interrogate data from people with epilepsy and compare these with control subjects. We therefore aim to explore further whether properties of the BNI derived from functional networks can be used as a marker in the clinical setting. We postulate that in some instances BNI may be able to distinguish between networks that appear similar when examined by traditional graph theoretic measures.

## Conflict of Interest Statement

The authors declare that the research was conducted in the absence of any commercial or financial relationships that could be construed as a potential conflict of interest.
